# A Case of Tussis Convulsiva Attended with Emphysema, That Terminated Fatally

**Published:** 1781-12

**Authors:** 


II. *A case of Tussis convulsiva attended with Emphysema, that terminated fatally. Communicated by* F. Swediar, M. D. *Physician iu London.* *Read* December 17, 1781.

THE following case of tussis convulsiva, on account of some singular circumstances that attended it, will perhaps not be deemed unworthy the attention of the Society.

A strong, healthy boy, about four years old, was attacked in June 1779 with symptoms of the hooping cough. The disease went on in its usual way, but at the end of a month the fits of coughing became uncommonly violent. Bleeding, emetics, a blister to the back, and a variety of antispasmodic medicines were tried without effect. At length a considerable tumour was perceived near the trachea, which upon examination was found to contain air. The patient's breathing became extremely difficult, and was attended with considerable wheezing. In less than twelve hours the whole body became emphysematous. The nature of the disease was now clearly ascertained, and the parents were informed of the danger of the case and of the necessity there was of puncturing the skin in order to discharge the air; but they obstinately refused refused to allow any such operation to be performed, and the child died the next day. The parents, who, like many other persons in the lower ranks of life, abounded with prejudices, would not permit the dead body to be inspected, so that we can only hazard a conjecture concerning the means by which the cellular membrane became so distended with air as to bring on a fatal emphysema. Is it not probable therefore, all circumstances considered, that the hooping cough, under which the child laboured, by its violence ruptured the ligamentous substance connecting the rings of the trachea, and so afforded an opening, through which the air gradually insinuated itself into the cells of the *tela cellulosa?*

